# Vinyl chloride accident unleashes a toxic legacy

**DOI:** 10.1016/j.ese.2023.100259

**Published:** 2023-03-01

**Authors:** Chengjun Li, Peng Gao, Riqing Yu, Huan Zhong, Mengjie Wu, Su Shiung Lam, Christian Sonne

**Affiliations:** Institute of Environmental Research at Greater Bay Area, Guangzhou University, Guangzhou, 510006, China; Department of Environmental and Occupational Health, and Department of Civil and Environmental Engineering, University of Pittsburgh, Pittsburgh, PA, 15261, United States; UPMC Hillman Cancer Center, Pittsburgh, PA, 15232, United States; Department of Biology, Center for Environment, Biodiversity and Conservation, The University of Texas at Tyler, Tyler, TX, 75799, United States; School of Environment, Nanjing University, Nanjing, China; Higher Institution Centre of Excellence (HICoE), Institute of Tropical Aquaculture and Fisheries (AKUATROP), Universiti Malaysia Terengganu, 21030, Kuala Nerus, Terengganu, Malaysia; Center for Transdisciplinary Research, Saveetha Institute of Medical and Technical Sciences, Saveetha University, Chennai, India; Department of Ecoscience, Arctic Research Centre (ARC), Aarhus University, Frederiksborgvej 399, PO Box 358, DK-4000, Roskilde, Denmark; Sustainability Cluster, School of Engineering, University of Petroleum & Energy Studies, Dehradun, Uttarakhand, 248007, India

A railroad accident on February 3, 2023, led to the release and combustion of 115,580 gallons, equivalent to over 437,000 L, of vinyl chloride monomer (VCM) in East Palestine, Ohio [1]. This monomer is used in polyvinyl chloride (PVC) production, and its burning produces additional toxins such as hydrochloric acid and lethal phosgene, known as a notorious chemical weapon during World War I [2]. Acute exposure to these chemicals causes immediate adverse effects on local ecosystems, including the deaths of wild and farmed animals and pets.

Significantly, in addition to volatilizing into the atmosphere, VCM can dissolve in natural waters or be adsorbed onto soil particles and remains stable in the absence of oxygen and sunlight [3]; thus, its residues can lead to long-term detrimental impacts on wildlife, including cancer and neurological disorders [4]. Acid rain induced by hydrochloric acid and associated acidification, compounded by other unknown and highly complex byproducts, harms plants, fish, amphibians, and other organisms, further deteriorating biodiversity and ecological biodiversity functions such as habitat destruction in the affected areas. The situation may be exacerbated, considering that the leaked chemicals and burning byproducts travel long distances in the air or rivers, impacting habitats outside the official evacuated zone.

One way to get around this is to establish comprehensive on-site sampling schemes and use remote sensing networks to monitor short- and long-term changes in abiotic and biotic components. This, together with in-depth impact assessments on nearby ecosystems by air and water transport modelling of contaminants, ensures real-time monitoring and effective mitigation in potentially affected regions [5]. In the short term, we need to rapidly identify the potential impacts on local ecosystems based on these measures, and ensure the swift and precise deployment of complementary mitigation strategies. These actions will help local communities to recover from the disaster.

However, bold moves are needed in the long term to avoid similar future catastrophes. An important aspect is a full collaboration among industries, authorities, and the scientific community to speed up phasing out VCM and find green and sustainable alternatives to PVC. Bio-based biodegradable plastics, such as fungi-derived fabrics upcycled from low-cost biomass materials, are promising substitutes for PVC products [6]. Incorporating such measures into existing multilateral environmental agreements, especially the Stockholm Convention, will reduce the risks of similar ecological disasters occurring.

**Figure:**
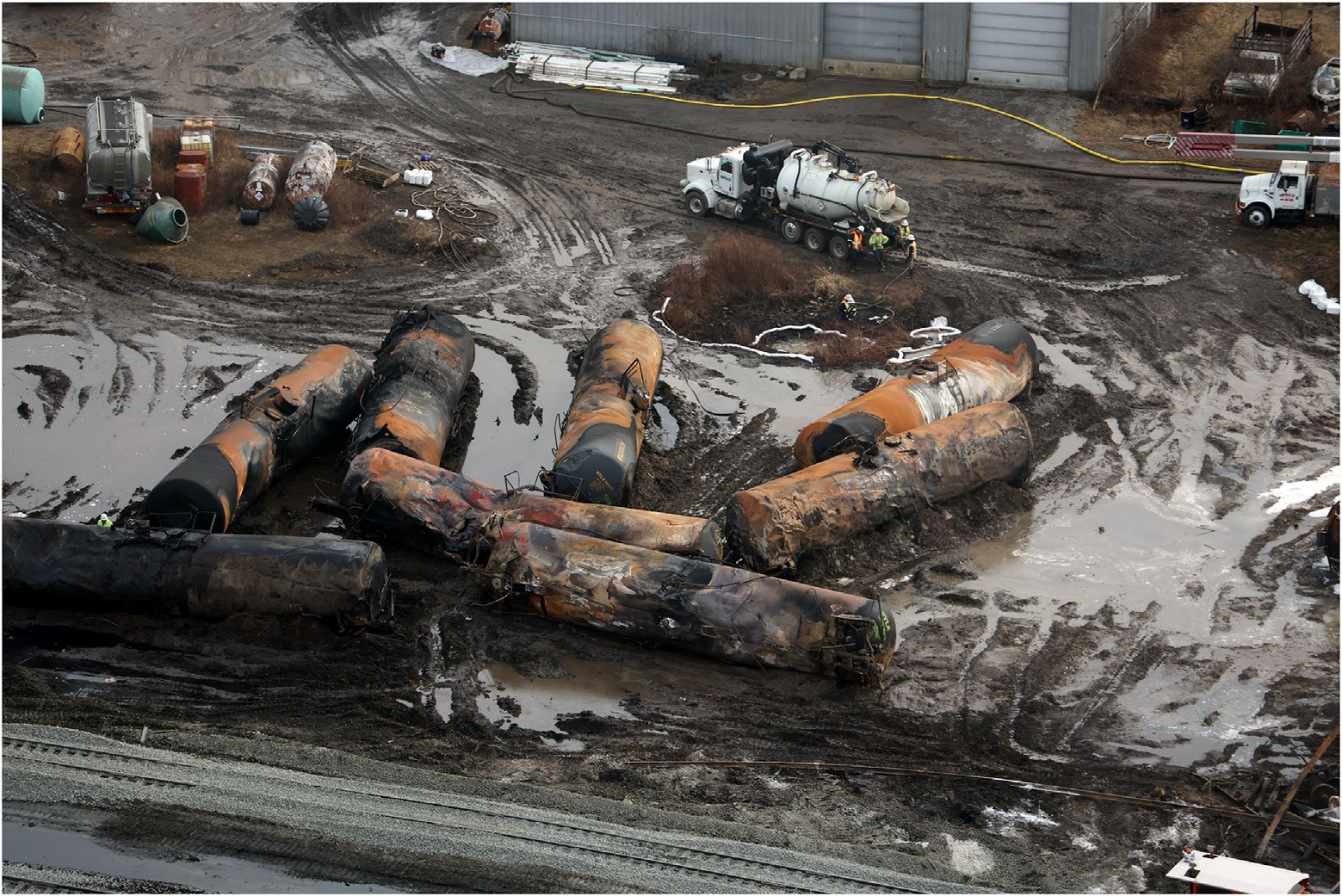
ALAMY/REF OY78766683
